# Immediate psychological effects of COVID-2019 in Emilia Romagna, Italy

**DOI:** 10.1192/j.eurpsy.2021.717

**Published:** 2021-08-13

**Authors:** I. Piretti, F. Ambrosini, R. Sant’Angelo

**Affiliations:** 1 Psychologist, Hr Specialist, Independent Researcher, Cesena, Italy; 2 Psychologist, Independent Researcher, Savignano, Italy; 3 Mental Health Department, AUSL ROMAGNA, Cesena, Italy

**Keywords:** Anxiety, stress, COVID-19, shelter in place

## Abstract

**Introduction:**

The epidemic caused by the SARS-CoV-2, which began in Wuhan city in December 2019, quickly spread to various countries around the world. On march Italy had already become the second country after China in terms of number of infections and deaths and Emilia Romagna was the second region in Italy by number of cases after Lombardy. On 11th of March 2020 through the “Stay at home” decree, the entire nation was sheltered-in-place (SIP).

**Objectives:**

Main objective is to understand immediate psychological effects on sheltered in place persons living in Emilia Romagna.

**Methods:**

This study is based on a cross-sectional online survey conducted anonymously in the period between the tenth and seventeenth day of SIP. We used Zung Anxiety Self-Assessment Scale, Insomnia Severity Index and Perceived Stress Scale 4. SPSS 21.0 was used for data analysis.

**Results:**

We collected data on 651 individuals. About 38% of the sample reported having sleep problems; 31% of the population has a minimal-moderate level of anxiety while 4% marked-severe; finally, 54% of the interviewees perceive a moderate level of stress while 31% high. The MANOVAs showed that anxiety is influenced by gender, age, level of education and occupational status. Greater levels of stress are shown by individuals who declared the fear of contracting the virus and the concern of financial loss.

**Conclusions:**

Our results could be used as a “psychological baseline” meanwhile the outbreak of COVID-19 is still ongoing. Despite the few days of SIP, we found the presence of a significant incidence and pervasive prevalence of psychological distress.

